# Dental Fees

**Published:** 1865

**Authors:** B. Wood

**Affiliations:** Albany, N. Y.


[For the Register.]

### DENTAL FEES.

BY B. WOOD, M. D.

Although this subject has become rather trite, it is one which will bear enforcing. "We" have already given "our views," but will venture to add a few remarks supplementary thereto.

As before stated, we find the profession virtually arranged in two classes on the Fee Question—the one contending for such compensation as shall warrant the exercise of the highest skill and the bestowment of the greatest benefit upon their patients; the other endeavoring to bring down the price to the lowest requirements of the masses. It may be admitted that, to a certain extent, self interest actuates both classes—at least, so far as to get the full value of their services. But while the one class is ambitious to render what will commend itself to liberal compensation, the study of the other is to do as little as possible at the smallest rates. The one caters to the highest bidder, the other to the lowest. When actuated by avarice, the one tends to extortion, the other to deception and imposition.

The inventive genius and latent skill of the American people are so fertile, and so adequate to any demand which will compensate the doing, that the public, by simply holding out adequate reward, could enlist the best talent in the land into service in the dental profession, and obtain for themselves the greatest benefits in the capacity of human skill. But the masses, and more especially the wealthy portion, having, from false training, habit, or sentiment, overspreading this country like an epidemic, come to value money for its own sake rather from the substantial benefits it will purchase, lend the weight of encouragement to those who, for the smallest fee, confer the least. Practically, they hold out a premium, not for the maximum but for the minimum skill, and, so doing, actually pay extortionate rates for miserable products.

But notwithstanding this, humanity, honor and honesty, clearly dictate which side our profession ought to take. This needs no argument; that the "best policy" leans the same way. Moreover, there is positive encouragement. A portion of the community, though it be a small portion, do seek the best services. They pay the rates necessary to secure them—the proof of earnest seeking; some not the most willingly, it is true, but who will nevertheless pay rather than forego the benefits. In addition to a just compensation, actual generosity might indeed be looked for on the part of wealthy patrons, in the way of bounties to unusual excellence in the profession, since artists, physicians, clergymen, men of letters and science frequently become the recipients of substantial appreciation of merit and fidelity, instances of which, in the case of dentists, are rarely known, however incalculable the benefit conferred. But then, on the other hand, the profession has not been signalized for the offer or bestowment of bounties for contributions to the resources of the art, conceiving a "vote of thanks" or a "medal" to be ample compensation for a life of toil and expenditure, and conceiving that such honor ought to render the recipients under enduring obligation to freely surrender up to the profession, or to whom the profession prefers to patronize, all pecuniary interest in their productions.

But let it suffice that there is a class of community who will and do concede a fair remuneration for the best services, (which really is all a manly independence will ask,) and that this class, amid the ebbs and flows of society, are sensibly on the increase. And when we consider that, purified by the scourge of war, elevated to nobler motives and impulses than the gratification of mere cupidity, ostentation and folly, schooled to self sacrifices, and habituated to acts of beneficence, the whole country has reached a higher stand point, from which wealth will be more generally recognized of value, chiefly as a means of procuring and dispensing real benefits, there is everything to encourage those in our profession who aim by their *good works* to confer the greatest good.

On the other hand there is no hope for the other class of operators. To whatever depth they may plunge in the scale of prices and quality of operations, a "lower depth yawns to receive them." The patronage they cater to in respect to *price* will never be satisfied even on this point. The search being for "cheap dentistry" nothing will be esteemed cheap except it be lower than the lowest. It is a habit or passion which strengthens as it is fed. It is a monomania which regards neither utility or beauty and often reveals itself in nothing but Dentistry—frequently confined to artificial teeth. The same individuals demand that, at any cost, their attire, the bonnet, the ribbons, the hat, cravat and collar, the hair and whiskers, curls, mustache, wig, etc., shall be becoming; their portraits, whether by pencil or photograph must be artistic and natural; but the *teeth*—"CHEAP."

All this from dint of effort to bring dentistry "within the reach of all," based on a low estimate of the nature of our calling. Why should it cost more to make and set a couple of plates of artificial teeth than to shoe a horse? Here, in Albany, a few resolutely stood up against the "outside pressure for the enormous fee of fifty cents for extracting a tooth, while the rest "pulled" for "a quarter." Why not at a quarter? A blacksmith will jerk out a horse nail for less money; and what is the difference? So the tooth puller lowers his figures, and will pull for still less by the quantity; and if there are new teeth to set will take out the old ones gratis, on the principle that the blacksmith considers the removal of the old shoe a part of the new shoeing.

It is a laudable competition prompted by benevolence to to the poorer classes.

But take some data from actual cases: A lady fluttering in silks and jewelry comes and has a tooth extracted. She is astounded at the fee—never heard of such a thing as as charging more than twenty-five cents. Fumbles for her money and finds she did'nt bring but that amount, not expecting it would be more ; promises to send the rest, (but never does), and sails out " *dissatisfied.*"

A ragged "darkey" comes next, pays his fifty cents with thanks, and goes rejoicing. A rich merchant, having two or three stores in operation, wants to know the price of re-pivoting a tooth—"has the tooth, all it needs is a hickory pin, and the old one drilled out." But the price appals him. Wants to know why your charges are so much higher than others; tells you the "customary price is a quarter;" has had the same tooth put in several times for that:—goes off to hunt up some one who wont extort. A poor decent looking man calls for a similar operation, pays the fee "saying it is reasonable enough."

Now there is no use of parleying with patients about the price; it loses time and gains nothing. Still worse is it to overrate the *cost of material* in extenuation of the fee, as many do. In the place of my former residence there was a pamphlet published wherein the author of it justified his underworking. He figured up the cost of gold used in plugs at 6¼ to 18¾ cts., and showed by mathematical calculation that he made abundant "profit" upon the cost, etc. When inquired of as to said cost, my reply was, it might be "6¼ cts." or five dollars, according to circumstances; I did not know and did not care; that we did not charge for material and did not look to the premium on it for remunerations; but based the fee upon the skill, time and attention devoted for the benefit of our cases. Upon the whole it resulted in a far juster appreciation of dental services than if I had elaborated about the number of sheets used, and all that.

I hold that the cost of material is of no more concern to the patient than the cost of the instruments with which we apply it, or than the cost of drugs in a physicians prescriptions. There is very properly a scale according to the kind of material, but this should be graduated by the time and skill involved in their use rather than upon estimates of the difference in cost.

These remarks refer to extremes, believing they will apply elsewhere, and that it is well to address the *common sense* of all, however low in the scale. To induce the meanest operators to charge better prices is so far an inducement to do better work.

ALBANY, N. Y.

